# Supplementary material for: Novel Populations of Mycobacterium smegmatis Under Hypoxia and Starvation: Some Insights on Cell Viability and Morphological Changes
Source: Microorganisms. 2024 Nov 10;12(11):2280. doi: 10.3390/microorganisms12112280 (PMC11596219; doi:10.3390/microorganisms12112280)
Supplement: Supplementary file 1 [file microorganisms-12-02280-s001.zip › microorganisms-3242965-supplementary.pdf]

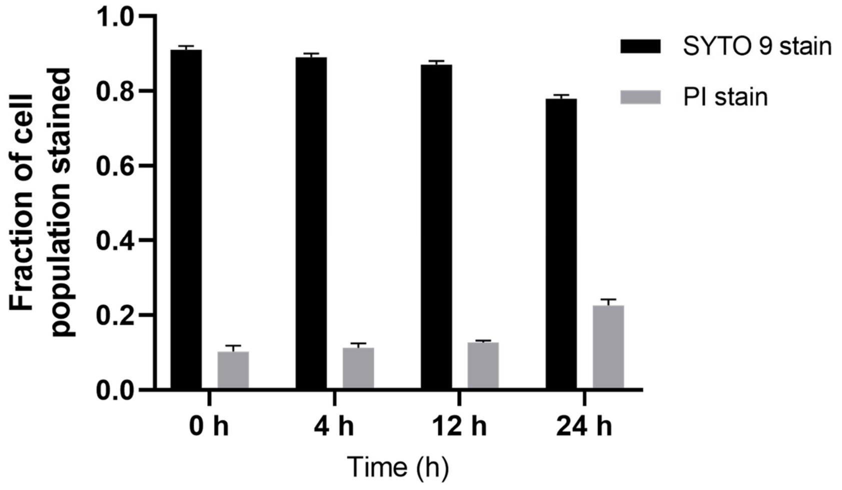

Supplementary Figure S1. Viability proportion of bacteria under starvation stress. The mycobacterial population was stained with SYTO 9 and propidium iodide (PI) to assess viability. The y-axis represents the total bacterial population. Over time, a clear decline in viability is observed, as indicated by the decreasing SYTO 9 staining, while the proportion of dead or compromised bacteria, marked by PI, increases. By 120 hours, SYTO 9/PI staining is no longer reliable due to the loss of bacterial membrane permeability, making it difficult to accurately measure viability at this time point. This suggests that prolonged stress leads to significant membrane compromise.
